# A pilot study of respiratory muscle training to improve cough effectiveness and reduce the incidence of pneumonia in acute stroke: study protocol for a randomized controlled trial

**DOI:** 10.1186/1745-6215-15-123

**Published:** 2014-04-12

**Authors:** Stefan Tino Kulnik, Gerrard Francis Rafferty, Surinder S Birring, John Moxham, Lalit Kalra

**Affiliations:** 1 Stroke Research Team, Department of Clinical Neuroscience, Institute of Psychiatry, King’s College London, PO Box 41, Denmark Hill, London SE5 8AF, UK; 2 Department of Respiratory Medicine and Allergy, School of Medicine, King’s College London, Chest Unit, Cheyne Wing, King’s College Hospital, Denmark Hill, London SE5 9RS, UK; 3 Division of Asthma, Allergy & Lung Biology, School of Medicine, King’s College London, Chest Unit, Cheyne Wing, King’s College Hospital, Denmark Hill, London SE5 9RS, UK; 4 Department of Respiratory Medicine and Allergy, King’s Health Partners, Chest Unit, Cheyne Wing, King’s College Hospital, Denmark Hill, London SE5 9RS, UK; 5 Stroke Research Team, Department of Clinical Neuroscience, Institute of Psychiatry, King’s College London, PO Box 41, Denmark Hill, London SE5 8AF, UK

**Keywords:** Respiratory muscle training, Prevention, Pneumonia, Stroke, Cough, Rehabilitation

## Abstract

**Background:**

After stroke, pneumonia is a relevant medical complication that can be precipitated by aspiration of saliva, liquids, or solid food. Swallowing difficulty and aspiration occur in a significant proportion of stroke survivors. Cough, an important mechanism protecting the lungs from inhaled materials, can be impaired in stroke survivors, and the likely cause for this impairment is central weakness of the respiratory musculature. Thus, respiratory muscle training in acute stroke may be useful in the recovery of respiratory muscle and cough function, and may thereby reduce the risk of pneumonia. The present study is a pilot study, aimed at investigating the validity and feasibility of this approach by exploring effect size, safety, and patient acceptability of the intervention.

**Methods/design:**

Adults with moderate to severe stroke impairment (National Institutes of Health Stroke Scale (NIHSS) score 5 to 25 at the time of admission) are recruited within 2 weeks of stroke onset. Participants must be able to perform voluntary respiratory maneuvers. Excluded are patients with increased intracranial pressure, uncontrolled hypertension, neuromuscular conditions other than stroke, medical history of asthma or chronic obstructive pulmonary disease, and recent cardiac events. Participants are randomized to receive inspiratory, expiratory, or sham respiratory training over a 4-week period, by using commercially available threshold resistance devices. Participants and caregivers, but not study investigators, are blind to treatment allocation. All participants receive medical care and stroke rehabilitation according to the usual standard of care. The following assessments are conducted at baseline, 4 weeks, and 12 weeks: Voluntary and reflex cough flow measurements, forced spirometry, respiratory muscle strength tests, incidence of pneumonia, assessments of safety parameters, and self-reported activity of daily living. The primary outcome is peak expiratory cough flow of voluntary cough, a parameter indicating the effectiveness of cough. Secondary outcomes are incidence of pneumonia, peak expiratory cough flow of reflex cough, and maximum inspiratory and expiratory mouth pressures.

**Discussion:**

Various novel pharmacologic and nonpharmacologic approaches for preventing stroke-associated pneumonia are currently being researched. This study investigates a novel strategy based on an exercise intervention for cough rehabilitation.

**Trial registration:**

Current Controlled Trials ISRCTN40298220

## Background

### Stroke-associated pneumonia

Pneumonia is a well-reported medical complication after stroke, in particular within the first weeks and months after the event. A recent review [1] identified 54 published studies reporting the incidence of pneumonia after stroke. These publications span from 1998 to 2012 and present data from intensive care, acute, and rehabilitation settings. The reported incidence of pneumonia is highest in intensive care patients (median, 27%; range, 4.1% to 56.6%), although ventilator-associated pneumonia may be a confounding factor in this setting. For acute stroke units, general medical wards, and rehabilitation units, the reported incidence of pneumonia ranges from 3.9% to 45%, with a median incidence rate of 7.4%. Studies differ in the criteria for the diagnosis of pneumonia, characteristics of study samples, and time periods of observation, and it may not be appropriate to compare studies directly. The most current and valid data available for the United Kingdom (UK) is from the national stroke audit for England, Wales, and Northern Ireland, which showed incidence rates of 16% in 2008 [2] and 13% in 2010 [3].

Pneumonia after stroke is associated with worse patient outcomes. Patients in whom pneumonia develops have an estimated twofold to sixfold increase in risk of death [4-15], are approximately 3 to 6 times more likely to have poor scores on various rehabilitation measures [6,7,11,12,16], stay in the acute hospital on average for 3 times longer than those without pneumonia, and also require higher levels of care after hospital discharge [4,7,9,12,14,17,18].

Swallowing difficulty (dysphagia) is one of the most frequently identified predictors of poststroke pneumonia [1,7,13,19-23]. Dysphagia is common in stroke, with an average incidence rate of 40% [24], although reported figures vary (14% to 94%) because of differences in sample selection and method and timing of swallow assessments [23,25-28]. Dysphagia is associated with an approximately twofold to threefold increase in risk of developing poststroke pneumonia [7,13,19-23]. This risk increases to fivefold to 11-fold with worsening severity of swallowing difficulty, the presence of aspiration (material entering the trachea past the vocal cords), and worsening severity of aspiration [23,29-31]. Several studies highlight the risk posed by silent aspiration (aspiration that occurs without triggering a protective cough) and the protection cough provides from aspiration pneumonia [32-34]. Some studies also demonstrate the association between weak or absent cough and higher incidence of aspiration in stroke survivors [35,36].

### Strategies for preventing stroke-associated pneumonia

To date, the single most successful strategy to prevent poststroke pneumonia has been the early detection of swallowing difficulty through routine swallow screening, followed by implementation of dysphagia-management strategies [1,9,21,37]. Various approaches to further reduce poststroke pneumonia rates are currently being researched. Pharmacologic approaches include the preventive administration of antibiotics [1,38], the use of ACE inhibitors to improve reflex cough sensitivity [39], and approaches targeting stroke-induced immunodepression [1]. Nonpharmacologic strategies include elevated body positioning to prevent aspiration [39] and reduction of oropharyngeal pathogens through intensive oral hygiene [1,39]. The present study investigates a novel nonpharmacologic approach, by using respiratory muscle training (RMT) to improve cough effectiveness and airway protection in acute stroke patients.

### Cough impairment in acute stroke

Cough is an important mechanism to protect from aspiration. It requires the coordinated activation of respiratory muscles (inspiratory and expiratory) and intrinsic laryngeal muscles. In a cough maneuver, inspiratory muscle action first causes air to be drawn into the lungs. Expiratory muscle contraction then creates a buildup of intrathoracic pressure against a closed glottis. Finally, a blast of air is released by rapid glottis opening, producing the characteristic cough sound and moving particles from the lungs toward and into the pharynx [40].

Stroke can adversely affect cough function. Peak expiratory cough flow (PECF), a measure of cough effectiveness, was found to be reduced by approximately one third in acute and chronic stroke patients when compared with healthy elderly subjects and normative values [41,42]. In detailed physiological studies of cough and respiratory muscle function, acute stroke patients were compared with matched healthy control subjects [43,44]. It was found that parameters of respiratory muscle strength (maximum inspiratory and expiratory mouth pressures, sniff pressure) and cough (PECF, gastric pressure during cough) in stroke subjects were reduced by one third to one half. This was demonstrated in voluntary and reflex cough. No difference between stroke and control subjects was found in the function of the intrinsic laryngeal muscles (glottis-closure phase during cough); or in respiratory muscle function when muscles were stimulated peripherally [43,44].

These findings demonstrate that a significant impairment of cough and respiratory muscle function occurs in acute stroke; that the impairment of cough function is likely related to respiratory muscle weakness, as opposed to dysfunction at the level of the glottis; and that the respiratory muscle weakness is related to the central component of the motor pathway (that is, the stroke lesion). Thus, an intervention targeting stroke-induced central respiratory muscle weakness may improve cough effectiveness and reduce the risk of stroke-associated pneumonia.

### Respiratory muscle training in stroke

Respiratory muscle training (RMT) aims to improve respiratory performance by loading the respiratory system beyond its usual level of functioning, thereby creating a training effect [45-48]. Much research on RMT has been conducted in healthy subjects, in athletes, and in clinical populations with primary respiratory problems. A small number of studies have investigated RMT in groups with neurologic conditions [49]. Two randomized controlled trials of inspiratory muscle training by using the threshold loading technique were conducted in chronic and subacute stroke patients [49-52]. Both trials demonstrated statistically significant improvements in inspiratory muscle strength and other physiological parameters for the RMT groups. Although the observed effect sizes were modest in absolute terms, these studies provide proof of principle that physiological improvement in stroke survivors is achievable through RMT.

### Aims and objectives

The present study investigates whether RMT is a worthwhile treatment approach to pursue for the reduction of pneumonia rates in acute stroke patients. The aim is to provide estimates on its magnitude of effect, acceptability, safety, and feasibility, and to inform about the value and design of a large clinical trial. The study objectives are (a) to determine the magnitude of effect of the intervention on cough generation, respiratory muscle strength, and incidence of pneumonia; (b) to explore the training duration, frequency, and intensity required to achieve improvement in cough flow and inspiratory and expiratory muscle strength; (c) to evaluate patient participation, acceptability of study procedures to participants, and concordance with training protocol; (d) to describe safety parameters and potential adverse effects of RMT in this patient group; (e) to describe characteristics of those patients most likely to gain from the intervention; and (f) to determine the relevance and feasibility of delivering RMT to acute stroke patients in a National Health Service (NHS) setting in the United Kingdom (UK).

## Methods/design

### Study design

**Figure 1:**
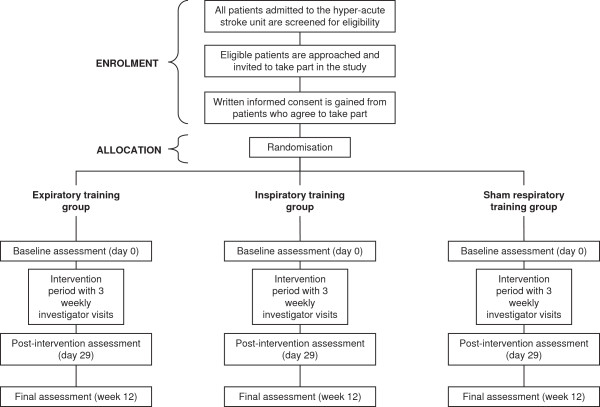
Flowchart of study design.

The study is a pilot study, designed as a single-blind randomized controlled trial with three study groups. Participants are randomized to receive inspiratory muscle training, expiratory muscle training, or sham RMT. After the baseline assessment, participants undergo a 4-week intervention period. Weekly investigator visits are conducted during the intervention period. The primary study end point is at the end of the intervention period (day 29). A final reassessment is conducted at 12 weeks after baseline. One investigator conducts all study procedures, including screening for eligibility, gaining consent, conducting study assessments, and introducing and assisting with the intervention. A summary of the study timeline and all data collected is given in Additional file 1. The participant flow through the study is described in Figure 1.

### Study setting

Acute stroke patients are screened for eligibility at one Hyperacute Stroke Unit (HASU) at one tertiary center in London, UK. Participants receive medical care and stroke rehabilitation according to the current standard of care. During the study period, participants may be discharged home or transferred to specialized stroke-rehabilitation units within the geographic area.

### Participants

The population of interest for this study comprises all patients admitted to hospital with an acute stroke, except for those with very mild stroke symptoms or without weakness. The inclusion and exclusion criteria reflect the nature of the study procedures, which require the participant to carry out volitional respiratory maneuvers with adequate technique; and the potential vulnerability of this acute patient group to extreme changes in intrathoracic pressures, which may be caused by respiratory maneuvers.

Inclusion criteria for the study are as follows:

– Age 18+ years

– Confirmed medical diagnosis of acute stroke (ischemic or hemorrhagic)

– Moderate to severe stroke impairment, defined as National Institutes of Health Stroke Scale (NIHSS) [53] score 5 to 25 and stroke-related weakness at the time of admission

– Within 2 weeks of stroke onset

– Ability to follow instructions and engage in study procedures

– Ability to give informed consent

Exclusion criteria are as follows:

– Signs of increased intracranial pressure on computed tomography (CT) scan

– Poorly controlled hypertension, defined as blood pressure higher than 180/100 at three or more occasions over the preceding 24 hours

– Myocardial infarction, angina, or acute heart failure in the preceding 3 months

– Pulmonary, neurologic (other than stroke), or orthopedic conditions adversely affecting the respiratory pump, including asthma and chronic obstructive pulmonary disease (COPD)

### Informed consent and ethical approval

Eligible patients are approached and invited to take part in the study. The study purpose and procedures are explained, taking into account any communication difficulties caused by the stroke. A study information sheet is provided. Patients are given at least 24 hours to consider the study. Only patients who have the ability to give informed consent are eligible. Patients who are willing to take part sign a written consent form. The study was granted ethical approval from the UK National Research Ethics Service (NRES) (Wandsworth Research Ethics Committee, study reference 10/H0803/32).

### Randomization, blinding, and treatment allocation

Sequentially numbered sealed envelopes containing the treatment allocation were prepared by a researcher unrelated to the study team. The randomization sequence was computer-generated. Block randomization (blocks of 12, containing four participants per study arm) was used to ensure even participant spread across the trial groups.

For each participant, the investigator opens the respective envelope after informed consent has been gained and immediately before conducting the study baseline session. Thereafter, the investigator is aware of the participant’s group allocation. Participants, caregivers, and healthcare professionals are unaware of group allocation throughout the study period.

### Intervention

The intervention consists of a respiratory muscle-strengthening exercise, by using the threshold RMT method [54]. A training stimulus is given by asking participants to breathe repeatedly in (inspiratory muscle training) or out (expiratory muscle training) through a commercially available hand-held resistance device (Threshold IMT, Threshold PEP; Respironics, Parsippany, NJ, USA). Participants are asked to train daily over a 4-week period. Every day, participants perform five sets of 10 breaths with the device, with resting periods of 1 minute between sets. The training resistance is set at 50% of the individual’s maximum mouth pressure (maximum inspiratory mouth pressure for the inspiratory training group, maximum expiratory mouth pressure for the expiratory training group). Maximum mouth pressures are reassessed weekly, and the training resistance readjusted accordingly. Participants in the sham training group are also given a training device, with the resistance set to an ineffectual 10% of maximum mouth pressure.

Participants are instructed in the correct training technique by the investigator during the baseline session, and training technique is reviewed weekly. To document concordance with training, participants keep a training diary, noting the frequency of training and any problems encountered.

### Study parameters

Additional file 1 gives an overview of the data collected at the respective time points. Peak expiratory cough flow (PECF) of voluntary cough at the end of the intervention period (day 29) is taken as the primary study outcome. This is in keeping with the clinical scenario of aspiration and resulting pneumonia. PECF is the parameter that indicates the speed of the air expelled from the lungs during the expulsion phase of cough. Voluntary cough PECF therefore reflects a person’s capacity to move aspirated particles from the lower airway back above the vocal cords and into the pharynx through a voluntary cough maneuver [55]. Secondary study outcomes are incidence of pneumonia, maximum mouth pressures, and peak expiratory cough flow of reflex cough at day 29.

Participants’ age, height, and weight at the time of hospital admission are recorded from clinical records.

Swallow safety is determined from the clinical swallow assessment, conducted routinely for patients admitted with acute stroke. This entails a swallow screen administered by a trained nurse. The swallow screen is conducted according to a clinical algorithm and includes observation of alertness, oro-motor function, and swallowing function. If concerns are raised, the medical team and the speech and language therapist are alerted. A clinical swallow examination by a speech and language therapist is performed, and swallow-management strategies are implemented. In specific cases, an instrumental assessment of swallowing function is performed.

For the purpose of the present study, the swallow is deemed unsafe if any precautionary instructions are issued for the participant, such as nil by mouth, recommendations of modified food textures, or specific swallowing techniques. The swallow is deemed safe, if the participant is allowed to swallow normal fluids and a normal diet without any explicit precautionary instructions.

The presence of pneumonia is determined from clinical records, from communication with the medical team, and from the participant directly.

The Nottingham Extended ADL Scale [56], a self-reported questionnaire, is used to assess participants’ functional independence in activities of daily living (ADLs).

All respiratory testing is conducted with the participant seated comfortably, or positioned sitting up in the hospital bed. Forced spirometry (forced expiratory volume at 1 second (FEV1), forced vital capacity (FVC), and peak expiratory flow (PEF)), is conducted according to international clinical standards [57], by using a portable spirometer (SpiroUSB; CareFusion, San Diego, CA, USA) and a bacterial filter (Spiroguard Standard; Air Safety Medical, Morecambe, England). A flanged mouthpiece (Rubber Flanged Mouthpiece MTH6400; CareFusion) is used to create an optimal mouth seal in the presence of orofacial weakness. Forced spirometry provides an assessment of participants’ lung function, and in particular of airway obstruction and restriction.

Respiratory muscle strength is assessed through measurement of maximum expiratory mouth pressure (MEP) and maximum inspiratory mouth pressure (MIP), according to international clinical standards [58]. A portable clinical device (MicroRPM; CareFusion) is used with a bacterial filter (Mouth Pressure Bacterial Filters FIL6050; CareFusion) and a flanged mouth piece (Rubber Flanged Mouthpiece MTH6400; CareFusion).

Cough flow measurements are conducted with a calibrated pneumotachograph system [59]. An on-site and an off-site measurement system are used. The on-site system is used for measurements at the primary study site. The off-site system is used for measurements outside the primary study site, for example at the neighboring stroke-rehabilitation unit or at participants’ homes. The on-site system consists of a Fleisch-type pneumotachograph (ID 4 cm, length 6 cm; PK Morgan Ltd, Rainham, England) connected to a face mask (Adult Face Mask, 8940 Series; Hans Rudolph Inc., Kansas City, MO, USA); a differential pressure transducer (MP45-14-871, range ± 2 cmH_2_O; Validyne Engineering, Northridge, CA, USA); a demodulator (CD15; Validyne Engineering); an analog-to-digital converter (PowerLab/16SP; ADInstruments Ltd, Oxford, England); and a laptop running LabChart data-acquisition software (LabChart Pro, version 7.2.2; ADInstruments Ltd).

For the off-site system, a different analog-to-digital converter (NI BNC-2110; National Instruments, Newbury, England) and laptop with data-acquisition software (LabView, version 5.1, National Instruments) are used. The on-site and off-site systems have the same performance characteristics: linear response from zero to 700 L/min in both directions of flow; frequency response of >20 Hz; and analog-to-digital sampling of 2 kHz. The systems are calibrated before each testing session by two-point calibration with a rotameter (InFlux OF1”S, 60 to 600 L/min flow; Techniquip Ltd, Taunton, England) at a reference flow of 500 L/min.

Voluntary and reflex coughs are assessed. For voluntary coughs, participants make repeated maximal cough efforts into a tight-fitting face mask, until five coughs of similar PECF are recorded. To elicit reflex coughs, escalating concentrations of capsaicin (0.49 to 1,000 μ*M*) are nebulized (UltraNeb U3000; DeVilbiss Healthcare Ltd, Tipton, England) and introduced into the face mask for 1 minute at a time, until the threshold is reached at which five bouts of reflex coughing are triggered. From the recorded cough-flow traces, the following parameters are derived for the five voluntary and five reflex coughs with the highest peak expiratory flow rates: peak expiratory cough flow (PECF), peak inspiratory cough flow (PICF), volume expired (VE), volume inspired (VI), glottis compression time, and cough-volume acceleration.

To monitor the safety of the intervention, blood pressure, heart rate, and blood oxygen saturation are measured before and after participants carry out RMT. Measurements are made by using a digital blood pressure monitor (UA-767 Plus; A&D Instruments Ltd, Abingdon, Oxon, UK) and a finger pulse oximeter (Onyx 9500; Nonin Medical Inc, Plymouth, MN, USA). In addition, the investigator notes any subjective discomfort reported during training. Adverse events are noted and reviewed throughout the duration of the study.

### Sample size

The initial sample-size calculation was based on observations from a cross-sectional study of acute stroke patients [44] and a one-way analysis of variance (ANOVA) model of statistical analysis [60,61]. For group sizes of less than 50, ANOVA assumes that data are normally distributed and that variances are equal. For the purpose of this sample-size calculation, these assumptions were made. With an estimated group standard deviation (SD) of 50 L/min for the main outcome measure (voluntary cough PECF at the primary end point), a sample size of 16 subjects per group gives the study 80% power to detect a 50 L/min difference between groups at the 5% significance level. Assuming an attrition rate of 20%, 20 subjects per study group are required, giving a total sample size of 60.

The sample-size calculation was reviewed after the first 40 participants completed their participation in the study. At this point, participant attrition was higher than anticipated (32%), and primary outcome data showed greater variability than initially assumed, with group SD ranging from approximately 100 to 250. Table 1 shows estimates of the required number of participants per group to give the study 80% power at the 5% significance level, according to different treatment-effect sizes and different group standard deviations. Taking into account these observed data, the decision was made to increase the study sample to 20 participants per group. This group size was selected to give higher statistical power should the data show large within-group variability. Also, it was considered a realistic recruitment target, given the observed rate of recruitment and the resources available. Taking into account a dropout rate of approximately 30%, the revised target sample is 90 subjects, whereby recruitment will be concluded early once 20 participants per group complete the primary study end point.

**Table 1:** Sample-size calculation

**Expected treatment effect (L/min)**	**Expected SD within group (L/min)**					
	**50**	**100**	**150**	**200**	**250**	**300**
50	15	58	125	250	400	600
100	5	15	35	60	95	125
150	<5	8	15	26	42	58
200	<5	5	10	16	24	34

Estimates of required number of participants per study group to give 80% power at the 5% significance level, according to different expected treatment-effect sizes and different expected group standard deviations (SDs) of the primary outcome (peak expiratory cough flow (PECF) of voluntary cough).

### Statistical analysis

Descriptive statistics will be used to present group characteristics at baseline; to compare study completers with participants who discontinued the study; and to compare participants with good training completion with those with poor training concordance. Training-safety data will be summarized descriptively and compared against pre-set safety parameters (standard safe ranges of vital parameters as used in clinical practice).

Inferential statistics will be used to evaluate the effect of treatment on the primary and secondary outcome parameters. The hypotheses under investigation are as follows: RMT (inspiratory or expiratory) is effective for improving voluntary cough effectiveness (voluntary cough PECF); RMT (inspiratory or expiratory) is effective for improving reflex cough effectiveness (reflex cough PECF); expiratory muscle training is effective for improving expiratory muscle strength (MEP); inspiratory muscle training is effective for improving inspiratory muscle strength (MIP); RMT (inspiratory or expiratory) is effective for reducing the incidence of pneumonia.

The sample-size calculation followed an ANOVA model of inferential statistical analysis. However, it is anticipated that the study sample will show considerable heterogeneity, in which case, an analysis of covariance (ANCOVA) model is more powerful in detecting a difference in study arms [62]. ANCOVA will be used to compare group means of voluntary and reflex cough PECF, MEP, and MIP at the primary end point (day 29). The following covariates will be adjusted for: gender, age, smoking, stroke severity (NIHSS score), training intensity, and a predictor variable for “missingness”, which will be determined through logistic regression. Fisher Exact test will be used to compare the incidence of pneumonia from baseline through week 4 between the three study groups. Adjustments for multiple testing will be made by using the Benjamini-Hochberg method [63].

Four approaches to dealing with missing values will be compared in a sensitivity analysis: intention-to-treat analysis, substituting missing values through predictive model-based imputation [64]; intention-to-treat analysis, substituting missing values through propensity score imputation [65]; intention-to-treat analysis, substituting missing values with baseline data; and complete case analysis, using only data from participants who completed the primary end point.

Further exploratory analyses may be conducted as thought appropriate, and any relevant findings will be reported accordingly.

The data-analysis plan has undergone some revision as the picture of the actual data observed became clearer with ongoing data collection, and it includes sensitivity and exploratory analyses. This flexible approach to data analysis is justified in this study, which is understood as a pilot project. Discovering what type of data can be expected in this population and comparing alternative statistical analysis approaches for these data falls within the aim of the project to inform the design of a larger clinical trial. However, the primary and secondary end points for the study have been clearly defined and have remained unchanged from the outset, and the results will be reported accordingly and in a transparent manner, regardless of “positive” or “negative” results. Although participant-level data will not be made publicly available, the data will be archived at the study center for 10 years according to regulatory requirements, where it will be available for scrutiny. Details of the statistical-analysis code will be included in the full study report.

### Dissemination

The research will be summarized in a technical report to the funding agency, the UK National Institute for Health Research (NIHR). This report will be in the public domain. The findings of the research will be disseminated locally, regionally, and nationally to professional and user groups through established research and service networks (for example, the South London Stroke Research Group, the UK Stroke Forum and the UK Stroke Research Network). A summary of findings written in lay language will be posted to study participants who wish to be informed about the study outcome. We will seek publication in peer-reviewed journals and presentations at international scientific meetings to reach the wider healthcare community. To ensure that the validity and scientific value of the study can be adequately evaluated by consumers of research, study reporting will follow the CONSORT [66,67] and CONSORT for nonpharmacologic interventions [68] guidelines. All dissemination outputs from the study require approval from the funding agency (UK NIHR) before publication.

## Discussion

### Strengths

To our knowledge, the present study is the first to explore respiratory muscle strengthening as a means to improving cough effectiveness and protecting from pneumonia in acute stroke. Although little research has been conducted in this area to date, several studies of good methodologic quality provide a physiological rationale and demonstrate the potential for physiological improvement through RMT in stroke.

The current study applies a method of cough flow measurement that is detailed and physiologically accurate. This method compares favorably with the use of portable peak flow meters, which have been used in other clinical studies in spite of uncertainty over their accuracy in measuring cough flow.

### Weaknesses

The methods for identifying the presence of swallowing difficulty and pneumonia in the current study may be regarded as a limitation. To minimize participant burden created by additional medical procedures, it was decided to take a pragmatic approach and observe clinical and self-reported information on swallowing function and pneumonia. A more-detailed instrumental assessment of swallowing, such as video-fluoroscopic examination of swallow, could be used to determine the degree of dysphagia and aspiration. Objective diagnostic criteria for pneumonia, including assessments of chest radiographs and blood results [69,70], could be used to support the detection of pneumonia.

A further limitation to the study is the lack of investigator blinding. The investigator is masked to treatment-allocation sequence, but is aware of group allocation from the point of randomization.

Although the clinical purpose of this research is to reduce the risk of pneumonia after stroke, the study uses voluntary cough flow as a surrogate primary outcome. This surrogate outcome was selected to minimize the required sample size. Designing the study according to the primary outcome, pneumonia, would require a considerably larger sample. For example, it could be assumed that both intervention groups would show a reduction in pneumonia rates from 15% to 7.5% compared with the control group. To give 80% power at the 5% significance level, this would require approximately 180 participants per group (using the χ^2^ test and not accounting for attrition).

The choice of voluntary cough flow as a surrogate primary outcome in this study was based on the physiological rationale to the research. Also, good evidence shows that reduced voluntary cough flow in stroke patients is associated with higher risk of aspiration, and therefore aspiration pneumonia [35,36]. To our knowledge, the direct relation between voluntary cough flow and pneumonia has not been investigated in stroke populations. The importance of cough flow may be inferred from populations with neuromuscular conditions, where reduced levels of voluntary cough flow indicate the need for assisted airway clearance [71]. The adequacy of voluntary cough flow as a surrogate measure for pneumonia will be assessed after the trial. It is likely that a proportion of study participants will develop pneumonia, and the association between voluntary cough flow and pneumonia will be assessed by using descriptive statistics or logistic regression analysis, as appropriate.

As described, the initial sample-size calculation was revised because of a higher-than-anticipated attrition rate. It is important to maximize participant retention in trials, to avoid conclusions that are biased through missing data [72]. It is anticipated that the present pilot study will indicate strategies to minimize participant attrition and missing data. So far, provision of transportation for participants free of charge and the option to bring testing equipment to the participant’s location have greatly improved participant retention. Further strategies will be explored by reviewing reasons for discontinuing the study and considering alternative methods of data collection, in an effort to prevent missing data.

In conclusion, in trialing RMT in acute stroke, the present study investigates a novel approach for preventing poststroke pneumonia. Poststroke pneumonia presents a multifaceted clinical problem, and different strategies for reducing incidence rates are currently being researched. The current study will provide valuable information on the potential validity and feasibility of this particular approach.

## Trial status

Recruitment to the study started in March 2011 and is expected to be complete by April 2014.

## Abbreviations

ACE: angiotensin converting enzyme; ADL: Activity of daily living; ANCOVA: analysis of covariance; ANOVA: analysis of variance; COPD: chronic obstructive pulmonary disease; CT: computed tomography; FEV: forced expiratory volume; FEV1: forced expiratory volume in 1 second; ID: internal diameter; IMT: inspiratory muscle trainer; MEP: maximum expiratory mouth pressure; MIP: maximum inspiratory mouth pressure; NHS: National Health Service; NIHR: National Institute for Health Research; NIHSS: National Institutes of Health Stroke Scale; NRES: National Research Ethics Service; PECF: peak expiratory cough flow; PEF: peak expiratory flow; PEP: positive expiratory pressure; PICF: peak inspiratory cough flow; RMT: respiratory muscle training; SD: standard deviation; UK: United Ki ngdom; USA: United States of America; VE: volume expired; VI: volume inspired.

## Competing interests

The authors declare no competing financial or nonfinancial interests in relation to the manuscript.

## Authors’ contributions

SK: design, data collection, analysis and interpretation, manuscript writing, and final approval of the manuscript. GR: design, data collection, analysis and interpretation, critical revision, and final approval of the manuscript. SB: design, data collection, analysis and interpretation, critical revision, and final approval of the manuscript. JM: design, critical revision, and final approval of the manuscript. LK: conception and design, data analysis and interpretation, critical revision, and final approval of the manuscript. All authors read and approved the final manuscript.

## Supplementary Material

Additional file 1 Study timeline and data collected at the respective time points. Click here for file
