# Supplementary material for: A pilot study of respiratory muscle training to improve cough effectiveness and reduce the incidence of pneumonia in acute stroke: study protocol for a randomized controlled trial
Source: Trials. 2014 Apr 12;15:123. doi: 10.1186/1745-6215-15-123 (PMC4021694; doi:10.1186/1745-6215-15-123)
Supplement: Additional file 1 — Study timeline and data collected at the respective time points. [file 1745-6215-15-123-S1.doc]

Additional file 1 Study timeline and data collected at the respective time points

|  | **STUDY PERIOD** | | | | | | | |
| --- | --- | --- | --- | --- | --- | --- | --- | --- |
|  | Enrolment | Allocation | Baseline assessment | Intervention period | | | Primary endpoint | Close-out |
| **TIME POINT** | (Within 14 days of stroke) | | Day 0 | Day 7 | Day 14 | Day 21 | Day 29 | Day 84 |
| **ENROLMENT:** |  |  |  |  |  |  |  |  |
| Eligibility screen | **X** |  |  |  |  |  |  |  |
| Informed consent | **X** |  |  |  |  |  |  |  |
| Allocation |  | **X** |  |  |  |  |  |  |
| **INTERVENTIONS:** |  |  |  |  |  |  |  |  |
| Expiratory muscle training |  |  |  |  |  |  |  |  |
| Inspiratory muscle training |  |  |  |  |  |  |  |  |
| Sham respiratory training (control group) |  |  |  |  |  |  |  |  |
| **ASSESSMENTS:** |  |  |  |  |  |  |  |  |
| Age, height, weight |  |  | **X** |  |  |  |  |  |
| Swallowing status |  |  | **X** |  |  |  | **X** | **X** |
| Presence of pneumonia |  |  | **X** |  |  |  | **X** | **X** |
| Voluntary and reflex cough flow measurements |  |  | **X** |  |  |  | **X** | **X** |
| Self-reported activities of daily living |  |  | **X** |  |  |  | **X** | **X** |
| Forced spirometry |  |  | **X** | **X** | **X** | **X** | **X** | **X** |
| Respiratory muscle strength tests |  |  | **X** | **X** | **X** | **X** | **X** | **X** |
| Vital parameters |  |  | **X** | **X** | **X** | **X** |  |  |
| Subjective symptoms during respiratory muscle training |  |  | **X** | **X** | **X** | **X** | **X** |  |
